# The Importance of P_bt_O_2_ Probe Location for Data Interpretation in Patients with Intracerebral Hemorrhage

**DOI:** 10.1007/s12028-020-01089-w

**Published:** 2020-09-11

**Authors:** Anna Lindner, Verena Rass, Bogdan-Andrei Ianosi, Alois J. Schiefecker, Mario Kofler, Paul Rhomberg, Bettina Pfausler, Ronny Beer, Erich Schmutzhard, Claudius Thomé, Raimund Helbok

**Affiliations:** 1 Neurological Intensive Care Unit, Department of Neurology, Medical University of Innsbruck, Anichstrasse 35, 6020 Innsbruck, Austria; 2 Department of Neuroradiology, Medical University of Innsbruck, Anichstrasse 35, 6020 Innsbruck, Austria; 3 Department of Neurosurgery, Medical University of Innsbruck, Anichstrasse 35, 6020 Innsbruck, Austria; 4 Institute of Medical Informatics, UMIT: University for Health Sciences, Medical Informatics and Technology, Eduard Wallnoefer-Zentrum 1, 6060 Hall, Austria

**Keywords:** Intracerebral hemorrhage, Neuromonitoring parameter, Critical care, Neurology

## Abstract

**Background/objective:**

Monitoring of brain tissue oxygen tension (P_bt_O_2_) provides insight into brain pathophysiology after intracerebral hemorrhage (ICH). Integration of probe location is recommended to optimize data interpretation. So far, little is known about the importance of P_bt_O_2_ catheter location in ICH patients.

**Methods:**

We prospectively included 40 ICH patients after hematoma evacuation (HE) who required P_bt_O_2_-monitoring. P_bt_O_2_-probe location was evaluated in all head computed tomography (CT) scans within the first 6 days after HE and defined as location in the *healthy brain tissue* or *perilesional* when the catheter tip was located within 1 cm of a focal lesion (hypodense or hyperdense). Generalized estimating equations were used to investigate levels of P_bt_O_2_ in relation to different probe locations.

**Results:**

Patients were 60 [51–66] years old and had a median ICH-volume of 47 [29–60] mL. Neuromonitoring probes remained for a median of 6 [2–11] days. P_bt_O_2_-probes were located in healthy brain tissue in 18/40 (45%) patients and in perilesional brain tissue in 22/40 (55%) patients. In the acute phase after HE (0–72 h), P_bt_O_2_ levels were significantly lower (21 ± 12 mmHg vs. 29 ± 10 mmHg, *p* = 0.010) and brain tissue hypoxia (BTH) was more common in the perilesional area as compared to healthy brain tissue (46% vs. 19%, adjOR 4.0, 95% CI 1.54–10.58, *p* = 0.005). Episodes of BTH significantly decreased over time in patients with probes in perilesional location (*p* = 0.001) but remained stable in normal appearing area (*p* = 0.485). A significant association between BTH and poor functional outcome was only found when probes were located in the perilesional brain tissue (adjOR 6.6, 95% CI 1.3–33.8, *p* = 0.023).

**Conclusions:**

In the acute phase, BTH was more common in the perilesional area compared to healthy brain tissue. The improvement of BTH in the perilesional area over time may be the result of targeted treatment interventions and tissue regeneration. Due to the localized measurement of invasive neuromonitoring devices, integration of probe location in the clinical management of ICH patients and in research protocols seems mandatory.

## Introduction

Despite improvements in the neurocritical care management of patients with hemorrhagic stroke over the past decades [1], intracerebral hemorrhage (ICH) is still a devastating disease associated with high rates of morbidity and mortality [2]. Multimodal neuromonitoring, including brain tissue oxygen tension (P_bt_O_2_) monitoring, provides insight into pathophysiologic changes of secondary brain injury, which in turn may open the opportunity to initiate interventions aiming to prevent additional brain damage [3]. As the assessment of P_bt_O_2_ is restricted to a small volume of brain tissue surrounding the tip of the probe, integration of probe location seems essential to optimize data interpretation and is therefore also recommended by guidelines and consensus statements [4, 5]. Primary placement of neuromonitoring catheters depends on the underlying disease entity, lesion location, and technical feasibility [4]. In patients with traumatic brain injury (TBI) P_bt_O_2_ probes should be placed in the lesioned hemisphere (hemisphere with higher lesion load, respectively) in order to detect brain tissue hypoxia in areas at highest risk for secondary injury [3, 6]. Absolute P_bt_O_2_ values may differ depending on the type of probe used [7] and probe location [5, 6].

Despite the assumed importance of neuromonitoring probe location in patients with ICH, no data describe the impact of probe location on absolute P_bt_O_2_ levels, their temporal dynamics and implications regarding outcome prediction. We hypothesized that multimodal neuromonitoring probe location largely influences P_bt_O_2_ levels and has an impact in the prediction of outcome after ICH. Therefore, we aimed to examine the influence of catheter location on P_bt_O_2_ levels and whether the prognostic association of brain tissue hypoxia (P_bt_O_2_ < 20 mmHg) with functional outcome depends on probe location.

## Methods

### Patient Population and Study Setting

**Fig. 1:**
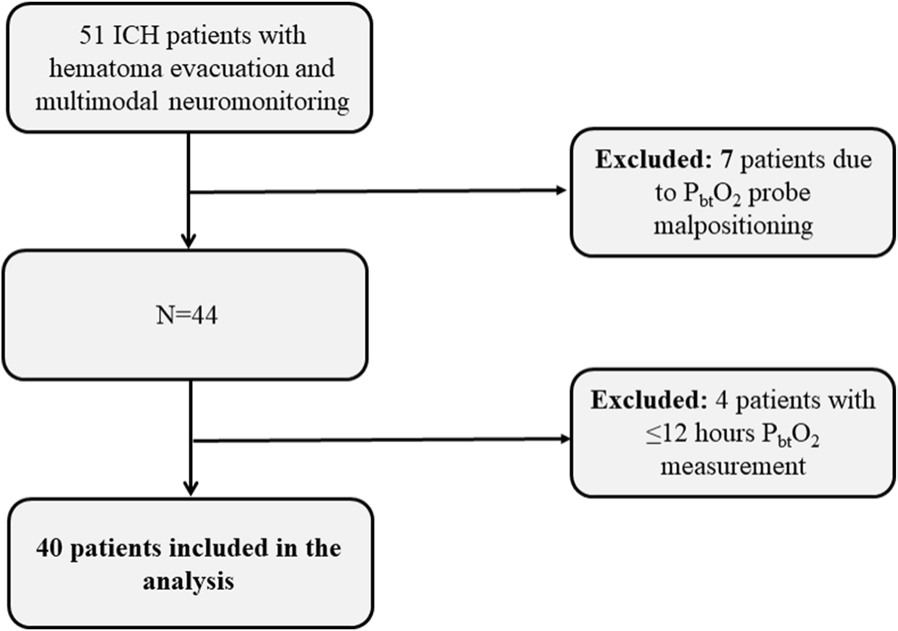
Patient selection. ICH = intracerebral hemorrhage

Prospectively recorded observational data of 51 consecutive spontaneous ICH patients fulfilling the inclusion/exclusion criteria for multimodal neuromonitoring admitted to the neurological intensive care unit (NICU) at a tertiary care center between 2011–2018 were studied. Neuromonitoring was initiated after hematoma evacuation (HE) in all patients and comprised assessment of brain tissue oxygen tension (P_bt_O_2_), intracranial pressure (ICP), and cerebral perfusion pressure (CPP). Patients were excluded if P_bt_O_2_ probes were dysfunctional (*n* = 7) or when recording time was less than 12 h (*n* = 4), leaving 40 patients eligible for the final analysis (Fig. 1). General inclusion criteria encompassed (1) admission with non-traumatic ICH diagnosed on cerebral imaging, (2) age ≥ 18 years, (3) hematoma evacuation, including the placement of invasive multimodal neuromonitoring as part of routine clinical care. The study was approved by the local ethics committee of the Medical University of Innsbruck (AN4088292/4.3, AM4091-292/4.6). Informed consent was obtained from all patients according to local regulations in accordance with the ethical standards as laid down in the 1964 Declaration of Helsinki. The reporting of data conforms to the STROBE guidelines.

### Patient Management and Data Collection

Clinical management of ICH patients conformed to current international guidelines set forth by the American Heart Association and European Stroke Association [8, 9]. The Glasgow Coma Scale (GCS), the Acute Physiology and Chronic Health Evaluation II (APACHE-II) Score [10] and the ICH Score [11] were used to assess the clinical and radiographic initial disease severity. Patients’ baseline characteristics, hospital complications, treatment interventions and outcomes were prospectively recorded in our institutional ICH database and confirmed in weekly meetings of the study team and treating neurointensivists. Continuous parameters (ICP, CPP, blood pressure (BP), P_bt_O_2_) were saved in our patient data management system (Centricity™ Critical Care 8.1 SP7; GE Healthcare Information Technologies, Dornstadt, Germany) and averaged (mean) over one hour. Up to the discretion of the treating neurointensivist, head computed tomography (CT) scans were performed, with the first CT scan obtained in all patients at admission, within the first 24 h and if clinically indicated. Hematoma volumes were calculated using the (ABC)/2 method on the admission CT and are given in milliliters (mL) by a blinded member of the study team [12].

Functional outcome was assessed by a study nurse blinded to the clinical course of patients using the modified Rankin Scale Score (mRS) 3 months after NICU discharge. In view of the high proportion of poor outcomes in patients with large size ICH, a mRS ≤ 4 was compared to a mRS between 5 and 6.

### Neuromonitoring and Probe Location

In this study, the decision to evacuate the hematoma was carefully evaluated in all patients by an interdisciplinary team including neurointensivists and neurosurgeons and performed by either surgical craniotomy (*N* = 32/40, 80%) or hemicraniectomy (*N* = 8/40, 20%). ICP was measured using a parenchymal ICP-probe (NEUROVENT-P-TEMP; Raumedic®, Helmbrechts, Germany) and P_bt_O_2_ by using a Licox® CC1.SB probe (Integra LifeSciences, Ratingen, Germany). None of the authors reposted any conflict of interest regarding the oxygen monitoring systems used. Whenever possible, multimodal monitoring probes were placed in the perihematomal brain tissue in order to monitor the tissue at highest risk for secondary brain injury. Probes were placed after HE without stereotactic support. Following the user manual, there is no requirement for a specific calibration as each P_bt_O_2_ probe includes a designated Smart Card. Additionally, FiO_2_ (FiO_2_ 100% for 5 min) challenges were performed daily to check the functionality of the probe. All patients were mechanically ventilated and received appropriate sedative medication as needed.

**Fig. 2:**
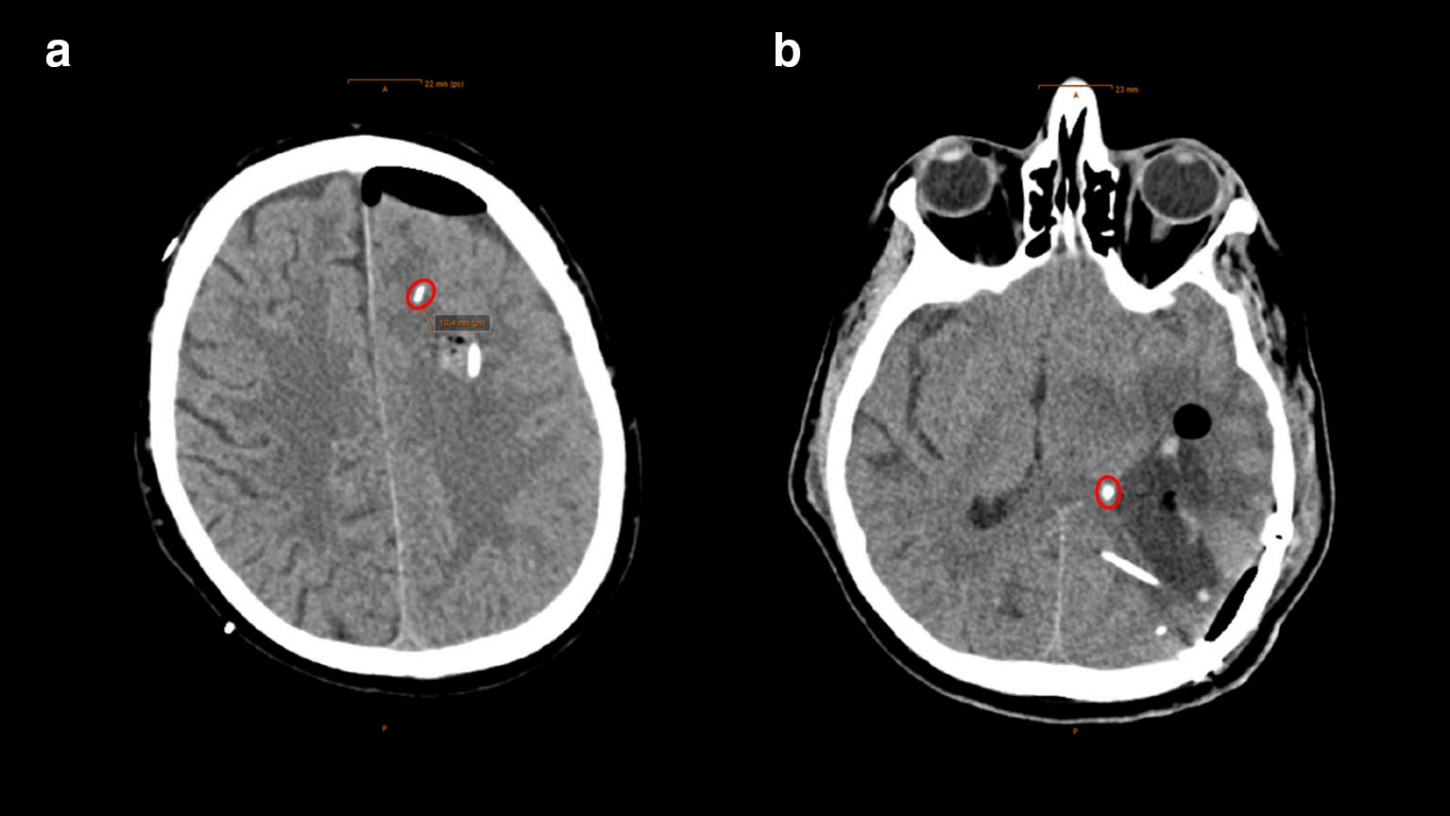
Figure shows examples for probe location in healthy brain tissue (**a**) and perilesional location (**b**) on axial computed tomography scans of the brain

The first CT scan after surgery was obtained within 24 h after probe insertion to evaluate probe location. Probe location was assessed by an independent neuroradiologist and AL on all available head CT scans within the first 6 days. If the tip of the P_bt_O_2_-probe was located within a 1-cm distance of a focal hyperdense lesion (parenchymal hemorrhage) and/or focal hypodense lesion (perihematomal edema, focal brain edema, ischemia), the location was defined as *perilesional,* which mostly reflected the perihematomal area. If there was no focal lesion within 1 cm of the tip, probe location was graded as *normal-appearing brain tissue*. (Fig. 2) Whenever there was direct contact with the hemorrhage (*intralesional location*), the data was excluded from the analysis. Brain tissue hypoxia (BTH) was defined as mean P_bt_O_2_ < 20 mmHg over one hour as considered in the Consensus Conference on Multimodality Monitoring in Neurocritical care and the current Seattle Consensus Statement and was treated in a stepwise multifactorial approach using an institutional protocol including a CPP target > 70 mmHg [4, 13, 14].

### Data Management and Statistical Analysis

Continuous variables (P_bt_O_2_, CPP, ICP, BP) were assessed for normality using the Kolmogorov–Smirnov test and reported as median and interquartile range [IQR] or mean and standard error of the mean (SEM), as appropriate. Statistical analysis was performed using SPSS 24 (IBM SPSS Statistics, version 24.0. Armonk, NY, USA). The acute phase (≤ 72 h) was compared with the subacute phase (73–168 h), for the assessment of temporal dynamics. To evaluate the impact of probe location on brain oxygenation, generalized estimating equations (GEEs) with the correlation matrix best fitting the data were used for time-series data analysis in order to account for repeated measurements within one patient. All multivariable models were corrected for predefined variables (age, GCS at admission and ICH volume at admission) based on their potential confounding influence on brain oxygenation and indicated appropriately [5, 15]. Adjusted odds ratios (adjOR) with 95% confidence intervals (CI) were calculated. In the same way, the associations with functional outcome were analyzed by using the dichotomized outcome factor as independent variable. Statistical significance was judged to a *p *value < 0.05. Graphical presentation was done using GraphPad Prism 7.

## Results

Patient characteristics, hospital complications and outcomes of 40 ICH patients are presented in Table 1. There was no difference in between patient groups (patients with probes in healthy brain tissue compared to those with probes in perilesional brain tissue). Patients were 60 [51–66] years old and had a median ICH volume of 47 [29–60] mL. All patients underwent hematoma evacuation, with a median of 9 [4–32] hours from hospital admission. Neuromonitoring probes were placed during surgery and remained for a median of 6 [2–11] days. In total, 89 head CT scans (2 [2–3] CT scans per patient) of the first 6 days after admission were analyzed. Median time to head CT scan after HE was 10 [5–14] hours. There were no neurosurgical complications recorded within the study time, including instant rebleeding or infarction related to the surgical procedure.

**Table 1:** Baseline characteristics, complications and outcome

Admission clinical findings	All patients (*N* = 40)	Healthy brain tissue location (*n* = 22)	Perilesional location (*n* = 18)	*p *Value
Female sex	12 (30)	5 (23)	7 (39)	0.271
Ethnicity, Caucasian	40 (100)	22 (100)	18 (100)	NA
Age in years	60 [51–66]	59 [48–67]	60 [58–66]	0.714
GCS at NICU admission	7 [3–13]	8 [3–12]	7 [3–13]	0.727
ICH Score (0–6)	2 [1–3]	2 [1–3]	2.5 [1–3]	0.801
Radiological characteristics				
ICH volume in mL	47 [29–60]	46 [27–54]	51 [39–76]	0.129
ICH volume > 30 mL	30 (75)	15 (68)	15 (83)	0.278
Intraventricular hemorrhage	13 (33)	7 (32)	6 (33)	0.919
Supratentorial origin	40 (100)	22 (100)	18 (100)	NA
Cortical	20 (50)	12 (55)	8 (44)	0.526
Basal ganglia	11 (28)	4 (18)	7 (39)	0.152
Thalamus	1 (3)	1 (5)	0 (0)	1
Subcortical white matter	8 (20)	5 (23)	3 (17)	0.635
ICH etiology				
Spontaneuos ICH	40 (100)	22 (100)	18 (100)	NA
Hypertensive	24 (60)	14 (64)	8 (44)	0.228
Cerebral amyloid angiopathy*	8 (20)	4 (18)	4 (22)	0.900
Arteriovenous malformation	3 (7.5)	2 (9)	1 (6)	0.447
Other**	5 (12.5)	0 (0)	5 (28)	NA
Surgical procedures				
Median admission to surgery time, in hours	8.5 [4–31]	7 [5–22]	11 [4–44]	0.842
Hemicraniectomy	8 (20)	5 (23)	3 (17)	0.635
Hydrocephalus requiring EVD placement	6 (15)	3 (14)	3 (17)	0.790
Residual hematoma volume after HE, in mL	7 [2–17]	5 [1–10]	10 [3–26]	0.104
Complications				
Rebleeding after HE	3 (7.5)	1 (5)	2 (11)	0.447
Secondary ischemia	5 (12.5)	4 (18)	1 (6)	0.255
Pneumonia	24 (60)	13 (60)	11 (61)	0.897
Ventriculitis	1 (3)	0 (0)	1 (6)	NA
Urinary tract infection	7 (8)	5 (23)	2 (11)	0.345
Sepsis	3 (7.5)	2 (9)	1 (6)	0.588
Outcome characteristics				
Length of NICU stay, in days	20 [14–35]	20 [14–31]	20 [16–39]	0.811
In-hospital mortality	4 (10)	1 (5)	3 (17)	0.233
3-month mRS				0.721
0	2 (5)	1 (4.5)	1 (5)	
1	1 (2.5)	1 (4.5)	0 (0)	
2	5 (12.5)	2 (9)	3 (17)	
3	4 (10)	2 (9)	2 (11)	
4	13 (32.5)	9 (41)	4 (22)	
5	11 (27.5)	6 (27)	5 (28)	
6	4 (10)	1 (5)	3 (17)	

*EVD* external ventricular drain; *GCS* glasgow coma scale score; *HE* hematoma evacuation; *ICH* score intracerebral hemorrhage score; *ICH* intracerebral hemorrhage; *mRS* modified Rankin scale; *NICU* neurologic intensive care unit

*Cerebral amyloid angiopathy was diagnosed according the Boston-criteria [36]: probable CAA with supporting pathological evidence in 5 patients and probable CAA in 3 patients

*HELLP-Syndrome (*n* = 1), cerebral vasculitis (*n* = 1), lobar hematoma of unknown etiology (*n* = 2), cerebral venous sinus thrombosis (*n* = 1)

Data are given in median [IQR] and counts (%). Univariate statistical analyses were performed using a linear model in GEEs variable

P_bt_O_2_-probes were located in healthy brain tissue in 18/40 (45%) patients and in perilesional brain tissue in 22/40 (55%) patients. Importantly, cerebral perfusion pressure (CPP) did not differ in patients with different probe locations both, during the early (*p* = 0.300) and delayed phase (*p* = 0.613) after HE. Episodes (> 1 h) of ICP ≥ 22 mmHg and mean number of administrations of osmotherapy were higher in patients with perihematomal probe location, although the results did not reach significant levels (*p* = 0.060 and *p* = 0.114, respectively).

In healthy brain tissue, mean P_bt_O_2_ levels were 29 ± 10 mmHg and in perilesional location 25 ± 12 mmHg (*p* = 0.144). Episodes of brain tissue hypoxia (BTH, P_bt_O_2_ < 20 mmHg) occurred in both groups (overall: 24% of monitoring time) and were slightly more common when P_bt_O_2_ was measured in the perilesional area (31% versus 18%, *p* = 0.074). (Table 2).

**Table 2:** Differences in neuromonitoring parameters and therapy intensity levels between patients with neuromonitoring probes in healthy brain tissue or perilesional location during the acute phase

Neuromonitoring parameters and therapy intensity level	Healthy brain tissue location (mean ± SD or percent (%))	Perilesional location (mean ± SD or percent (%))	*p* value
Patients, *n*	23 (57)	17 (43)	
Average P_bt_O_2_ (mmHg)	29 ± 10	21 ± 12	0.022
Episodes (> 1 h) of brain tissue hypoxia	18	31	0.005
Intracranial pressure (mmHg)	11 ± 6	15 ± 8	0.041
Episodes (> 1 h) of intracranial hypertension (ICP > 22 mmHg)	5	14	0.060
Cerebral perfusion pressure (mmHg)	71 ± 10	69 ± 10	0.300
MAP (mmHg)	82 ± 10	84 ± 11	0.623
pCO_2_ (mmHg)	40.1 ± 3.6	39.7 ± 3.6	0.471
Mean administration of osmotherapy	1.7 (in 9 patients)	3.6 (in 10 patients)	0.114
Hemoglobin (g/l)	114 ± 16	110 ± 13	0.400
Midazolam (mg)*	1077 ± 697	1766 ± 888	0.287
Noradrenalin (mg)*	32 ± 21	36 ± 18	0.684

This table shows values collected in the acute phase (< 72 h)

*IQR* interquartile range, *MAP* mean arterial blood pressure; *P*_*bt*_*O*_*2*_ brain tissue oxygen tension, *pCo*_*2*_ partial pressure of carbon dioxide, *SD* standard deviation. Univariate statistical analyses were performed using a linear model in GEEs variable.

*Mean dosage per patients of sedatives or catecholamines during the acute phase.

### Trends in Brain Tissue Oxygen Tension Over Time in Relation to Probe Location

**Fig. 3:**
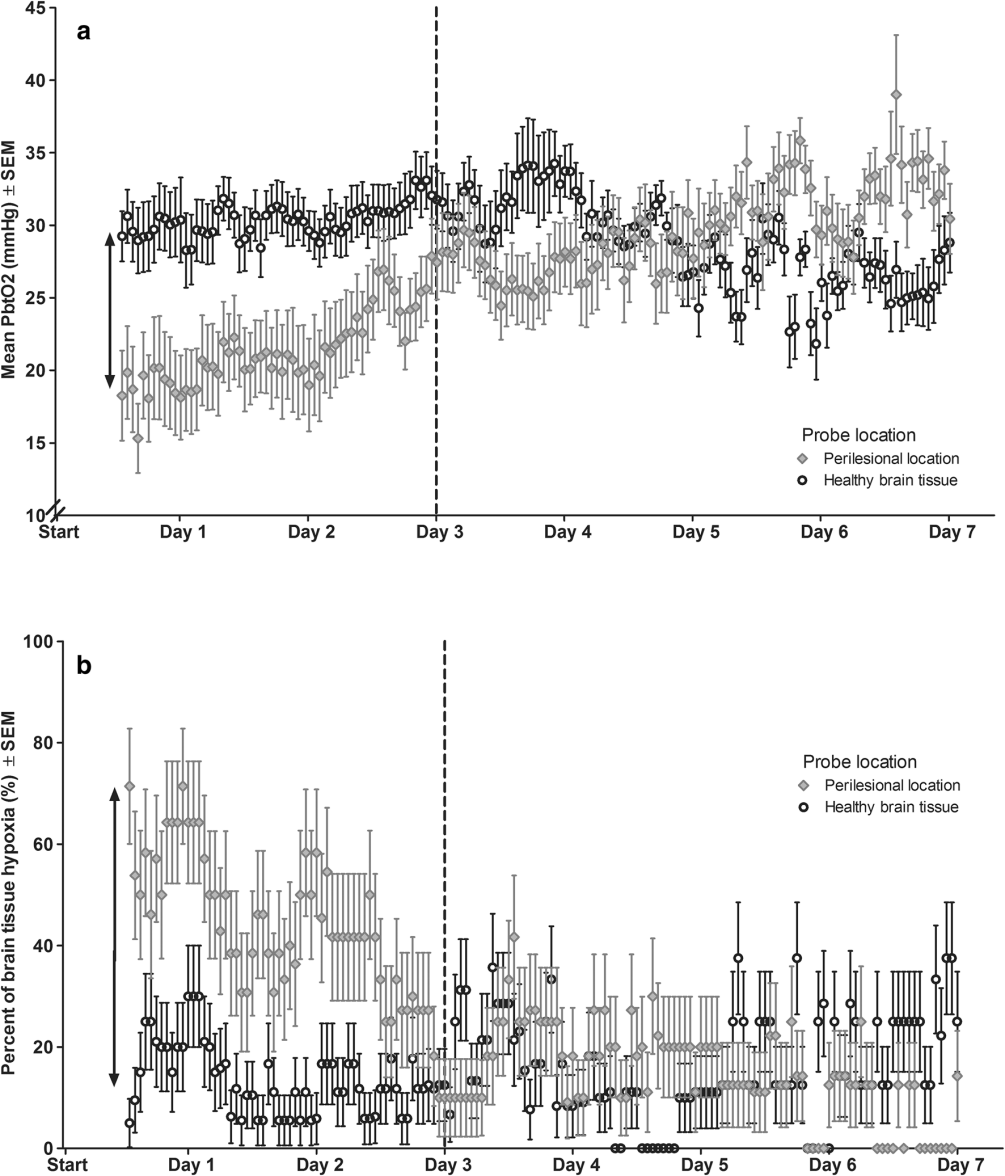
Mean P_bt_O_2_ values (Panel A) and difference between the occurrence of brain tissue hypoxia (Panel B) in normal-appearing brain tissue or perilesional area. The dashed line separates the acute (≤ 72 h) from subacute phase (73–167 h)

In two patients, we found a shift of catheter tip location from healthy brain tissue to perilesional tissue (*n* = 1) or from perilesional location to intralesional placement (*n* = 1); in all other patients probe location remained in the same position during neuromonitoring time. In the early phase after surgery (≤ 72 h), P_bt_O_2_ levels were significantly lower in comparison to the subacute phase (21 ± 12 mmHg vs. 29 ± 10 mmHg, *p* = 0.010; Fig. 3, Panel A). In the early phase, BTH was more common in the perilesional area compared to healthy brain tissue (46% vs. 19%, adjOR 4.0, 95% CI 1.54–10.58; *p* = 0.005, Fig. 3; Panel B) even after adjusting for admission GCS Score, age and ICH volume. Episodes with BTH significantly decreased over time in patients with probes in perilesional location (from 42% on day 0 to 14% on day 6, *p* < 0.001). Episodes with BTH remained stable when probes were located in normal appearing area (*p* = 0.485). There was no impact of probe location on BTH during the subacute phase (BTH: 16% vs. 17%, adjOR 1.1, 95% CI 0.29–4.1, *p* = 0.884).

## P_bt_O_2_-Probe Location and Outcome

Patients severely disabled or dead (mRS ≥ 5) 3 months after ICH showed a trend toward lower overall P_bt_O_2_ values (25.5 [19.7–32.8] mmHg vs. 28.8 [20.6–36.1] mmHg, *p* = 0.201) and a higher incidence of BTH than patients with better outcome (26% vs. 23%, *p* = 0.336). Multivariable analysis revealed a significant association between BTH and major disability or death (adjOR 6.6, 95% CI 1.3–33.8, *p* = 0.023) when probes were located in the perilesional brain tissue. Moreover, patients severely disabled or dead showed a trend toward lower mean P_bt_O_2_ values, when probes were placed in perilesional location (23 [23–29] mmHg vs. 27 [17–36] mmHg), compared to patients with better outcome (adjOR 0.92, 95% CI 0.83–1.01, *p* = 0.086). Neither BTH nor P_bt_O_2_ were associated with functional outcome when the probe was placed in healthy brain tissue (*p* = 0.807 and *p* = 0.465, respectively). All models were adjusted for admission GCS score, ICH volume and age. (Table 3).

**Table 3:** Association between probe location and major disability or death*

	Perilesional location			Healthy brain tissue		
	adjOR	95% CI	*p* value	adjOR	95% CI	*p* value
Admission GCS	1.7	1.1–2.5	0.013	1.3	0.92–1.74	0.148
Brain tissue hypoxia	6.6	1.3–33.8	0.023	1.2	0.37–3.61	0.807

*Adjusted for ICH volume and age

*ICH* intracerebral hemorrhage; *GCS* glasgow Coma Scale; Brain tissue hypoxia is defined as brain tissue oxygen tension (P_bt_O_2_) ≤ 20 mmHg; “Major disability and death” is defined as a 3-month modified Rankin Scale Score (mRS) ≥ 5. Statistical analyses were performed using a linear model in GEEs

### Discussion

To the best of our knowledge, this is the first study analyzing the impact of P_bt_O_2_-probe location on longitudinal P_bt_O_2_ levels in patients with spontaneous ICH. The main findings of this study are that (1) episodes of brain tissue hypoxia are more frequent in the perilesional location and (2) that lower P_bt_O_2_ levels were only associated with 3-month functional outcome when the probe was located close to a lesion.

Invasive multimodal neuromonitoring can provide insight into metabolic, electrographic and oxygenation changes in distinct areas of the brain. [5, 16] It is crucial to integrate catheter location for data interpretation although an individualized treatment concept has not been developed so far [13, 16]. The impact of catheter location has been described in patients with TBI and SAH [17–20], but has not been systematically analyzed in ICH patients so far.

P_bt_O_2_ values reflect the balance between oxygen delivery, consumption, tissue diffusion and extraction [21] and can be influenced by various factors such as cerebral autoregulation status [22, 23], neurovascular coupling [24], CO_2_ levels [25], probe location [5, 6] as well as factors that contribute to an increased consumption of local oxygen such as fever [26], spreading depolarizations [27] and epileptic seizures [28].

It is recommended to insert the tip of the P_bt_O_2_ catheter in the most affected hemisphere targeting “perilesional” brain tissue in TBI patients [5, 6]. In patients with diffuse brain injury, neuromonitoring probes should be inserted in the non-dominant frontal lobe. In patient with SAH, catheters should be placed in the brain tissue of watershed of the aneurysm bearing vessel [29]. For ICH patients, there is no recommendation regarding the optimal placement of P_bt_O_2_ probes. Due to the localized measurement, integration of probe location in the clinical management of ICH patients seems mandatory. It remains unclear if brain oxygenation parameters are representative for the whole brain (most likely in the normal appearing brain tissue on head CT-scan) or data derived from the perilesional brain tissue should be integrated into clinical decision making [6, 30]. In the current study, we could confirm higher rates of BTH in the perilesional brain tissue in the early phase which is in line with previous reports [31]. In ICH patients, the perihemorrhagic area is most vulnerable to secondary brain damage due to the proinflammatory response and other secondary injury cascades [32]. Interestingly, we found that BTH significantly decreased over time in our patients which could reflect a regeneration process of the perihematomal area after ICH [33] or the impact of our P_bt_O_2_-based protocol to prevent brain tissue hypoxia [14]. This would support the hypothesis that the perihematomal area may be amenable to treatment. Importantly, there was no significant difference indicating a more aggressive treatment in patients with perilesional probes as compared to those with probes in healthy appearing tissue within the acute phase. Still, we observed more often episodes of intracranial hypertension in patients with perihematomal probe location, although the results did not reach significant levels. Either way, monitoring of P_bt_O_2_ in the perihematomal area may open up a novel treatment target in individualized patients in order to prevent further brain injury. Still, our data should be confirmed prospectively to quantify the effect of implementing a P_bt_O_2_ guided treatment protocol. Improvement in P_bt_O_2_ levels in the perilesional area over time has previously been described in TBI patients [5, 6, 14, 33].

Deleterious effects of BTH are well known in neurocritical care patients [19, 34]. Recently, a Phase III study proved that the time spent in BTH can be significantly reduced when applying a P_bt_O_2_-based protocol in TBI patients. Although underpowered to detect an impact on outcome, descriptive outcome analysis seemed promising [18]. In these studies, the location of the P_bt_O_2_ probe was not integrated. It is important to mention that the association between BTH and outcome has not been thoroughly studied in ICH patients. We found an overall incidence in BTH of 24%. Interestingly, BTH was only associated with poor functional outcome when measured in the perilesional area, which is consistent with the findings in TBI patients by Ponce et al. [5] and data derived from a swine model [35]. Our results support the placement of P_bt_O_2_ probes in the perihematomal tissue, since P_bt_O_2_ values obtained in perilesional tissue bear greater prognostic potential than levels measured in normal-appearing tissue. This may be explained by the less frequent occurrence of BTH episodes in healthy brain tissue. Moreover, it might also be that healthy parenchyma is less sensitive to BTH, and therefore, there is a need for different thresholds for BTH than in perilesional location.

There are some limitations that warrant consideration. First, we present a retrospective analysis of prospectively collected data. Accordingly, we cannot specify causalities but only associations. Moreover, the patient cohort was a selected group of poor-grade ICH patients requiring HE and invasive neuromonitoring. Consequently, we determined a mRS ≤ 4 to dichotomize outcome. Still, our results describe clinical practice of P_bt_O_2_ monitoring in poor-grade ICH patients and may help to better understand data derived from neuromonitoring. Third, there is a limitation grading P_bt_O_2_ catheter location by using less sensitive CT scans rather than MRI. As a result, there may be undetected minor ischemic or hemorrhagic lesions that may have affected P_bt_O_2_ levels. Although we found that P_bt_O_2_ values might be even more relevant in the perilesional tissue for outcome prediction, further studies are needed to investigate the pathophysiologic cascades leading to BTH in the early phase of ICH. Despite the fact we aimed for a perilesional probe location in our patients by placement after HE and by tunneling the devices, this was only achieved in 18/40 patients. There are insufficient data to support an imaging guided procedure, which would facilitate probe location. Even if probe positioning may not be improved, interpretation of absolute P_bt_O_2_ levels strongly depend on probe location. Moreover, our data suggest that low P_bt_O_2_ levels sincerely matter in the perihematomal brain tissue. Still, more studies are needed to test the hypothesis that a more aggressive treatment to increase P_bt_O_2_ levels in the perihematomal area would ameliorate outcome in these patients. Furthermore, in the current study we used a single probe to monitor the oxygen profile in ICH patients and separated the groups depending on neuroimaging results revealing probes either in the perihematomal brain tissue or in normal appearing brain tissue. Our results are supported by a porcine model where 4 concurrent P_bt_O_2_ probes were placed in different regions. P_bt_O_2_ values were strongly influenced by the distance from the site of focal injury with the lowest values in the perihematomal area [35]. A recommendation for individual patients may not be given based on our results. Still, our data confirm the brain tissue hypoxia in the perihematomal area is important in outcome prediction.

## Summary/Conclusions

This observational study indicates that BTH was more common in the perilesional area and has prognostic implications. Furthermore, BTH was more pronounced in the early phase in ICH patients. Our data suggest that P_bt_O_2_ values obtained in perilesional tissue bear greater prognostic potential than levels measured in normal-appearing tissue. In addition, these results support the hypothesis that BTH in the perilesional area improves over time and is potentially amenable to treatment.
